# Gene expression of behaviorally relevant genes in the cerebral hemisphere changes after selection for tameness in Red Junglefowl

**DOI:** 10.1371/journal.pone.0177004

**Published:** 2017-05-08

**Authors:** Johan Bélteky, Beatrix Agnvall, Per Jensen

**Affiliations:** AVIAN Behavioural Physiology and Genomics Group, IFM Biology, Linköping University, Linköping, Sweden; Embrapa, BRAZIL

## Abstract

The process of domestication in animals has led to alterations in behavior, physiology and phenotypic traits, changes that may be driven by correlations with reduced fear of humans. We used Red Junglefowl, ancestors of all domesticated chickens selected for either high or low fear of humans for five generations to study the effects of selection on gene transcription in the cerebral hemisphere, which is heavily involved in behaviour control. A total of 24 individuals from the parental generation as well as from the fifth selected generation were used. Twenty-two genes were significantly differentially expressed at *p* < 0.05 after false discovery rate (FDR) correction. Those genes that were upregulated in the low fearful animals were found to be involved in neural functions. Gene ontology and pathway analysis revealed enrichment for terms associated with behavioural processes. We conclude that five generations of divergent selection for high or low tameness has significantly changed gene expression patterns in the cerebral hemisphere in the Red Junglefowl population used here, which could underlie a range of changes in the domestic phenotype.

## Introduction

Domestication is the process in which a wild population of animals through selective breeding is formed into thriving in captive environments under human control, with several alternative mechanisms involved in the first phases of the domestication process [1, 2]. The process induces a plethora of phenotypic changes in behaviour, physiology and morphology, mediated by genetic and epigenetic mechanisms [3]. Domestic animals generally tend to become more docile, change foraging and social behaviors; furthermore, coloration changes, reduced brain size and an earlier onset of sexual maturity are also typical consequences of domestication [4, 5]. The occurrence of similar traits in unrelated domestic animals is usually referred to as the domesticated phenotype [6], and despite the prevalence and research on the it, there is very little knowledge regarding the underlying genetic mechanisms [7].

Previous attempts at experimentally domesticating wild animals in order to study the domestication process have been performed on, e.g., silver foxes and rats [8, 9]. The fundamental theory behind these domestication attempts is that the central trait driving other parts of the domesticated phenotype is the selection against fear of humans, which is a necessary firsts step for any successful domestication [10]. Many of the changes associated with domestication such as color phenotypes, size differences and behavioural alterations could then be a byproduct of the initial selection for tameness. One persuasive example concerns the silver foxes mentioned above, which after half a century of selection against fear has produced animals with many of the behavioral, physiological and morphological traits associated with the domesticated phenotype [9]. In a similar study, lab rats were bred for high and low aggression during 64 generations [11]. The two strains showed extreme differences in behaviour during handling and also differed in several neuroendocrinological and morphological traits [12, 13].

Here, we utilize Red Junglefowl (*Gallus gallus*), the ancestor of today’s domestic chickens. The domestication of chickens began approximately 8000 years ago in Southeast Asia, the native origin of the Red Junglefowl [14, 15]. Today’s modern chicken breeds show high diversification stemming from a long history of selective breeding, and with intense recent breeding programs for production traits such as egg production and meat yield. The Red Junglefowl is an excellent model to study early effects of domestication, since they are relatively easy to breed in captivity and unlike foxes and rats, the species has in fact been domesticated in historic time. Our previous studies have shown that when selecting on divergent levels of fear of humans only, correlated changes in social dominance, weight, plumage condition, basal metabolism and hypothalamic gene expression are observed after only two to six generations [16–18]. Further studies of transcriptional effects in the brain may increase our understanding of the genetic mechanisms underlying the cascade of phenotypic effects caused by changes in tameness, and this is the purpose of the present study.

Previous research looking at the effects of domestication on gene expression has mainly focused on the cerebral hemisphere. Albert et al [19] compared five different species of wild and domestic mammals in order to look for patterns common to all the domesticates. The results, however, indicated specific changes for each domestication case rather than general changes common for all events. The cerebral hemisphere was chosen based on its involvement in social cognition [20] and ease of dissection in the various species. The Red Junglefowl provides an excellent case study for changes during early domestication, as previously reported patterns all stem from the study of mammals. The inclusion of a bird could answer questions pertaining to possible conserved mechanism in Amniota. Based on previous studies we decided to perform a similar comparison of gene expression profiles in the Red Junglefowl, selected for divergent levels of tameness. In this experiment, the anterior cerebral hemisphere from high and low fearful chicken from the fifth generation of selection (S5), and their original parents (P0) was used in order to study gene expression changes related to the selection for high or low fear of humans. The aim of the experiment was to investigate whether the selection for a behavioural trait over a number of generations affects gene expression in a behaviorally relevant tissue.

## Material and methods

### Ethical note

The experiments were carried out in accordance with regulations for animal experimentation, and were approved by the Linköping Animal Ethics Committee, license no 122–10.

### Animals and sampling

An outbred parental starting generation (P0) was generated by crossing two Red Junglefowl populations with different zoo origins, and from this we bred lines for increased vs reduced fear of humans during five generations. For a detailed breeding scheme and housing conditions, see previously published work [16, 21]. In short, birds in the parental generation were tested in a standardized fear-of-human test. This test is thoroughly described in [16]. Briefly, chickens were tested one and one in an arena, where a human approached it according to a standardised protocol and the behaviour of the bird was scored throughout the test. Based on the behavioural scores, two subpopulations were created with either the most or least fearful birds. This was the basis for the two selection lines, and in addition an unselected population was kept and randomly bred in each generation as a control. All three strains were hatched and reared in mixed groups in the same environments in order to standardize the rearing conditions. About 50 animals per selection line were reared for each new generation. The environment was kept as constant as possible between generations. Birds were all raised in the same buildings, using the same equipment and feed.

For gene expression analysis, we studied animals from the parental (outbred and unselected) generation (P0), and from the fifth selected generation (S5). From the parental generation only birds which were used as parents for the high and low selection lines, i.e., those with extreme scores in the behavioural test, were used. In the S5 selection lines, birds were randomly selected regarding fear scores, but with consideration to family structures to avoid sampling closely related individuals. Due to the limitations in the number of available animals it was not possible to sample chickens from different ages. All sampling was therefore made at the time when the entire generation was culled.

A total of 24 individuals were used for the analysis: eight P0 (two males and two females from the high fearful parental group and equal amount from the low fearful parental group), and 16 individuals from generation S5 (two male and two female from the unselected group, three male and three female from the high fearful group, and three males and three females from the low fearful group). Birds were killed at the age of 350 days by decapitation, and brains were carefully dissected into several parts, two of those being the left and right cerebral hemisphere, of which the right one was used for the gene expression analysis in the study. The brain parts were snap frozen in liquid nitrogen within ten minutes of decapitation, and then moved to -80°C freezers for long-term storage.

### Sample preparations and microarray analysis

Upon thawing of samples, RNA from each of the 24 samples was isolated and used to synthesize labelled cRNA for subsequent microarray analysis. Two out of the 24 arrays failed quality control and was removed from the analysis.

RNA was extracted from the right anterior cerebral hemisphere using an Allprep RNA/DNA kit (Qiagen, Germany) following the manufacturer’s instructions. In short, approximately 30 mg frozen tissue was homogenized with 600 μl Buffer RTL Plus using a FastPrep® -24 (MP Biomedicals, USA) before transferring the homogenate to an AllPrep DNA spin column. The filtered RNA flow-through was mixed with 150 μl chloroform by vortexing before addition of 80 μl Proteinase K (20mg/mL) and 350 μl 100% ethanol. The mixture was then transferred to an RNeasy spin column. The RNA was cleaned via centrifugations with RPE Buffer, DNase I, Buffer FRN and pure ethanol in order to produce a clean yield. For elution of the RNA into a 1,5 ml microcentrifuge tube, 30 μl of RNase-free water (Ambion, USA) was used. Quantitative analysis of the RNA samples were made using a NanoDrop® ND-1000 (Thermo Scientific, USA), followed by quality control based on RNA Integrity Numbers (RIN) with a Bioanalyzer® instrument (Agilent Technologies, USA).

Once all RNA samples passed the quality control, they were converted to Cyanine 3-CTP labelled cRNA using one-color Low Input Quick Amp Labeling Kit (Agilent Technologies, USA), following the manufacturers’ protocol. In short, 200 ng RNA per sample was prepared in tubes, and mixed with 5000-fold diluted Spike-in Mix providing a positive control for the hybridization. Samples were mixed with T7 Primer and denatured at 65°C for 10 min followed by a 5 min incubation on ice. A master mix containing reagents for cDNA synthesis was added to all samples followed by a two hour incubation at 40°C. The enzymes were inactivated by a 15 min incubation at 70°C before addition of a transcription master mix. The samples were then incubated for two hours at 40°C in order to synthesize complementary RNA whilst simultaneously labeling it with Cyanine 3-CTP. All samples were then purified using a NucleoSpin® RNA kit (Macherey-Nagel, Germany) following the manufacturers protocol. After quantification of the samples, they were hybridized to SurePrint G3 Custom 8x60K microarrays (Agilent Technologies, USA) overnight. All arrays were then scanned on an MS200 Microarray scanner (Roche NimbleGen, USA), and data was extracted via the Feature Extraction Software v12.0 (Agilent Technologies, USA).

Microarray data has been uploaded to Array Express (http://www.ebi.ac.uk/arrayexpress/browse.html) under accession E-MTAB-4741.

### Statistical analysis

Expression data was analyzed using R (http://www.r-project.org) and Bioconductor (www.bioconductor.org). The normalization and preprocessing, and subsequent analysis of expression data was performed using the Bioconductor package *limma*. Two arrays failed quality controls and were excluded before the normalization process. Differentially expressed (DE) genes (probesets on the array are consistently referred to as genes in the results and discussion) were identified using a linear model approach [22]. For both gene expression analysis and gene ontology, Benjamini & Hochberg [23] was used for FDR correction of *p*-values. For cluster analysis and generating heat maps, the hierarchical clustering function in Genesis software v 1.7.6 [24] was applied. Samples were separated into two groups; high and low fearful S5, and P0 and unselected S5, before applying the clustering function. Extraction of gene ontology terms, KEGG pathways and disease associations were done using gene symbols obtained in biomaRt [25], run against the human genome in the online tool WebGestalt [26] in order to increase possible hits.

## Results

When comparing expression between the high and low fearful groups in the P0 generation, no significant differences were found, indicating that the initial division of the animals into different fear groups from the larger population was not associated with gene expression differences between the groups. Neither was there any significant difference between the P0 generation as a whole and the S5 unselected group, implying a low likelihood of genetic drift and that the unselected group constitutes a valid control.

**Fig 1:**
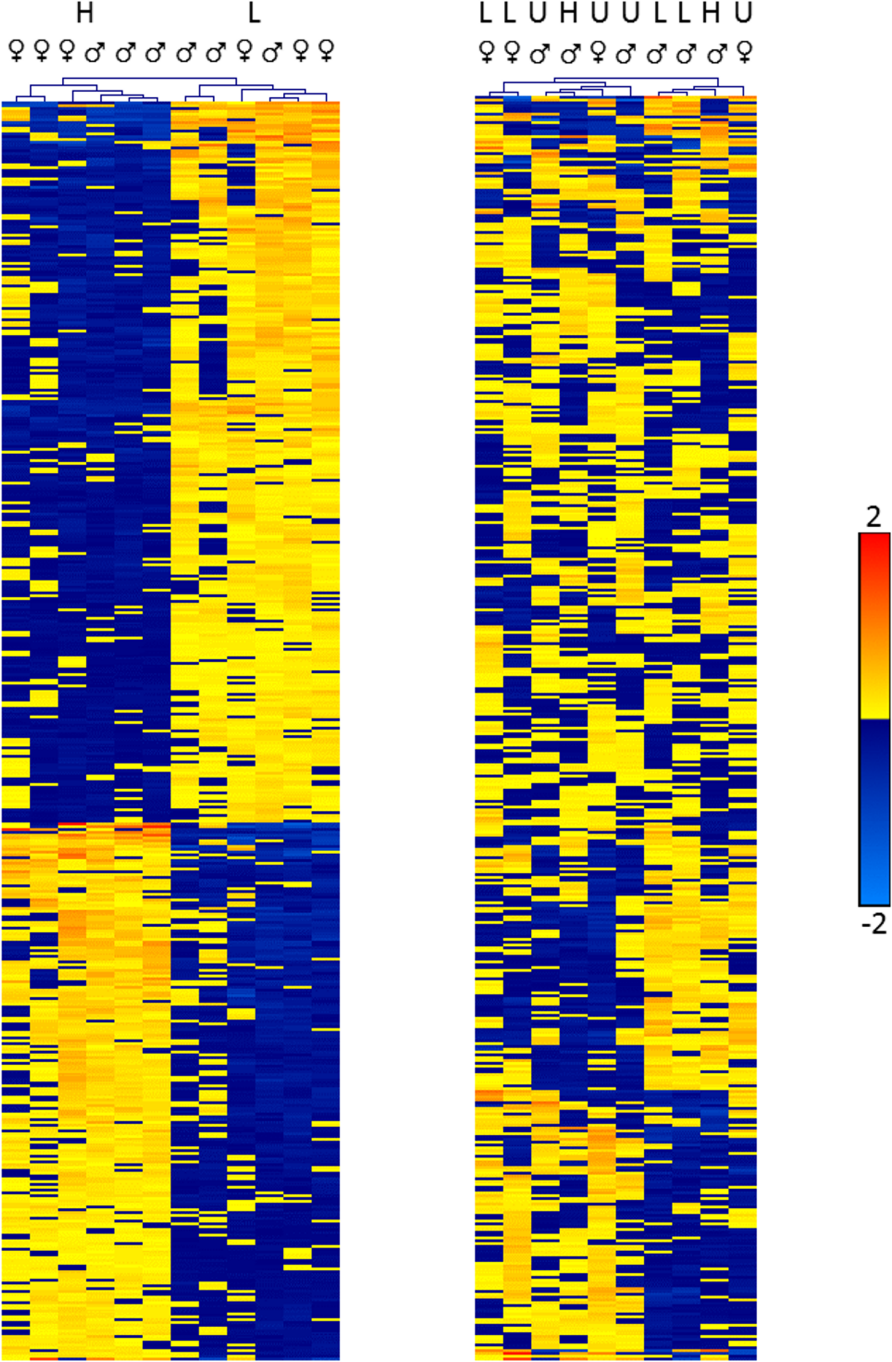
Gene expression clustering analysis. Heat map showing the relative expression levels for 417 genes significantly differently expressed (unadjusted *p*-value < 0.01) between (a) high and low fearful S5 animals and (b) P0 and unselected S5 animals. Columns represent individual samples while rows represent genes from the microarray analysis, structured through hierarchical cluster analysis (average linkage). H, high; L, low; U, unselected.

In the S5 generation, neither high nor low fearful birds differed significantly from the unselected group. However, comparing the high and low fear birds in the S5 resulted in 417 genes with significantly different expression between groups (S1 Table). When adjusting for multiple testing, the number of genes still being significantly differently expressed dropped to 22, as presented in Table 1. Hierarchical clustering analysis of the 417 significant gene expression differences in S5 identified two clusters in the experimental samples, corresponding to the high and low fearful groups (Fig 1). Analysis of the same genes in P0 and unselected S5 showed no clustering by fearfulness, indicating that the differentially expressed genes in S5 are the result of selection during the five generations and not an inherent difference from the P0 population split.

**Table 1:** Significantly differentially expressed genes between high and low fearful animals in generation S5. Twenty-two genes were significantly differentially expressed at p < 0.05 after adjusting for multiple comparisons. Gene names are provided for those genes where annotations exist, chromosomal location of the probe, logarithmic fold change (logFC), and both unadjusted and adjusted p-values.

*Gene*	*Chromosome*	*Start (bp)*	*logFC*	*p (unadj)*	*Adj p*
*RBPMS*	10	478309	-1,3791	7,48E-09	0,00013
*RFT1*	12	1137009	-0,6973	2,02E-08	0,00018
*ENSGALG00000019306*	1	54705220	-0,8885	2,14E-07	0,0013
*SPAG4*	25	691949	1,4495	6,03E-07	0,00183
*NEU3*	1	200653895	-0,9109	5,31E-07	0,00183
*ENSGALG00000022845*	2	107527960	-0,9180	5,3E-07	0,00183
*C9H2orf72*	9	16227550	-0,8865	1,5E-06	0,00389
*CDAN1*	5	28042117	-1,1607	3,33E-06	0,00757
*DYDC1*	6	5484912	1,2159	1,1E-05	0,02237
*TOR3A*	8	6551984	-0,3930	1,49E-05	0,02693
*RNF43*	19	564499	-0,3698	1,63E-05	0,02693
*DCLK2*	4	33892014	0,6561	1,91E-05	0,02898
*ENSGALG00000016237*	1	115815831	0,4708	2,42E-05	0,03217
*MBTPS2*	1	122556164	-0,5356	2,47E-05	0,03217
*CCDC103*	27	1259550	-1,1050	2,65E-05	0,03220
*ADPRHL2*	23	4462263	0,4896	3,28E-05	0,03735
*SNAP23*	5	27881718	-0,5364	3,85E-05	0,04125
*MRPS18A*	3	32021644	0,8156	4,88E-05	0,04484
*TNFSF15*	17	2959452	0,3152	4,52E-05	0,04484
*STK38L*	1	70241341	-0,5090	5,91E-05	0,04805
*PM20D2*	3	78525921	-0,7893	5,85E-05	0,04805
*ZSWIM5*	8	21668917	0,6877	6,06E-05	0,04805

In order to determine the direction of the expression changes, the expression level (normalized signal strength from the microarray) of the 22 significant genes in S5 were compared with the same expression levels in the parental high and low fear groups. Six genes (*RBPMS*, *RFT1*, *TOR3A*, *SNAP23*, *C9orf72* and *ENSGALG00000022845*) were upregulated in the low fearful group compared to P0, whilst two genes (*SPAG4*, *MRPS18A*) were upregulated and one (*STK38L*) downregulated in the high fearful group. For the remaining 13 genes, expression levels were not significantly different in any of the selection lines compared to P0.

Gene ontology (GO) analysis of the 22 significant genes showed no clear results with respect to enrichment of similar types of function. However, a GO analysis of the 417 genes with unadjusted p-values below 0.01 showed a significant enrichment of terms connected to cellular components such as cytoplasm (GO:0005737; adjP = 8.93e-06), basement membrane (GO:0005604; adjP = 3.09e-02) and mitochondrion (GO:0005739; adjP = 9.6e-03), and the molecular function protein binding (GO:0005515; adjP = 8.2e-03). The biological functions neural crest cell development (GO:0014032) and differentiation (GO:0014033) showed significant enrichment, but not after FDR correction. KEGG pathway analysis yielded enrichment of a few terms such as metabolic pathways (ID:01100; adjP = 0.0029), insulin signaling (ID:04910; adjP = 0.0496), neurotrophin signaling (ID:04722; adjP = 0.0166), axon guidance (ID:04360; adjP = 0.0441) and long-term potentiation (ID:04720; adjP = 0.0441). Disease association analysis was carried out (based on human homologues and their associations with human disorders) and yielded top disease terms mental (DB_ID:PA447208; adjP = 9.69e-05) and mood disorders (DB_ID:PA447209; adjP = 0.0015), bipolar disorder (DB_ID:PA447199; adjP = 0.0009), depression (DB_ID:PA447278; adjP = 0.0009) and mitochondrial disease (DB_ID:PA447172; adjP = 0.0019).

We further compared the gene lists obtained for any overlap with relevant previous studies of domestication or tameness induced gene expression differences. Out of the 417 genes with unadjusted p values below 0.01, 39 genes overlapped with a similar list based on hypothalamic gene expression in the same birds [18]. Out of these, five were significantly differentially expressed after FDR correction in the cerebral hemisphere and five in the hypothalamus. Three of them were significant in both tissues: *SPAG4*, *RFT1* and *ENSGALG00000016237*. GO analysis for the 39 genes did not reveal any significant terms. Additionally, 11 genes out of the 417 overlapped with domestication related selective sweeps found previously in chicken [27], two genes overlapped with genes affected in a sample of domesticated mammals [19] and two genes overlapped with a list of genes significantly differentially expressed in hypothalamus in a comparison between Red Junglefowl and modern domesticated White Leghorn chickens [28]. For a complete list over genes overlapping with other studies, see S1 Table. Considering all the comparisons described, no gene from the present experiment was found overlapping with lists from more than one study.

## Discussion

The results presented here show that gene expression profiles in the cerebral hemisphere in two different selection lines of Red Junglefowl diverge after five generations of selection for either high or low fear of humans. The functions of the differentially expressed genes were not immediately associated to fear behaviour, but for a number of genes did reflect neurological relevance. The results suggest that the significant gene expression changes after only five generations of divergent selection for tameness could be part of an underlying genetic mechanism generating several correlated changes related to the domestic phenotype.

The experimental population has been extensively studied previously [16–18, 21]. The high and low fearful strains show differences in several behaviors, with foraging and conspecific aggression increasing in low fear animals. Several other traits, relating to metabolism, size and growth, and plumage condition also differ between the two selection lines after only a couple of generations. Fear and stress is mediated through the hypothalamus, and examination of hypothalamic gene expression data from the same animals used in this study indicated a moderate number of gene changes between high and low fearful birds in the fifth selected generation, with no obvious function in relation to tameness [18]. Instead, the genes were mainly involved in immunological and reproductive processes, suggesting possible routes for the correlated phenotypic effects in these birds. The present study aimed to further investigate the genetic differences underlying the phenotypic changes in a tissue more closely associated with behaviors important in domestic animals, i.e., the cerebral hemisphere.

The mechanisms behind the well-documented cascade of uniform phenotypic changes in different domesticated animals are not understood. Two main theories have been proposed, both focusing on pleiotropic effects of genes targeted during the focused selection. It should also be noted that the two theories are by no means mutually exclusive. The first was proposed by the farm-fox experiment founder Belyaev, who termed it “destabilizing selection” and suggested that selection for reduced fearfulness would disrupt central neuroendocrine pathways which in turn would cause a cascade of changes as the system is reformed [10]. The other theory is more recent, and suggests the involvement of neural crest cells (NCCs) [7, 29]. The theory suggests that modifications in migration of NCC during embryology cause many aspects of the domesticated phenotype. It is not within the scope of this study to differentiate between these two (or other) theories of causal effects, but it is worth noting that we observed GO terms related to neural crest cells, suggesting that the chicken could be a valid model for studying this further.

Just as previously shown in hypothalamic gene expression data from the same population, the initial outbred P0 generation did not show any significantly different expression of genes between the high and low fear group [18]. The P0 generation as a whole group did not seem to differ from the unselected group in S5. Neither did significantly different genes between high and low fearful birds in S5 show the same tendencies of grouping in P0, as indicated in Fig 1. What more is that P0 and unselected S5 did not group separately, meaning that the unselected group did not diverge from P0 throughout selection. This indicates that the observed differences between the high and low fear selection lines were caused by the selection process and not from incidental genetic drift. It is clear that drift is probably a major factor contributing to the domestication process at large, but the significant changes between the high and low fearful S5 groups in the present experiment are most likely an effect of the selection imposed.

Among the 22 identified significant genes presented in Table 1, nine showed changes specific to either selection group (i.e. being either up- or down-regulated in comparison with P0). Six genes differed in the low fear group, and out of these one is un-annotated and one is annotated as chromosome 9 ORF72. For the four genes with annotation, *RBPMS*, *RFT1*, *TOR3A*, *SNAP23*, previous studies have them associated with neurological disease or function. The gene *RFT1* (protein RFT1 homolog) encodes for an enzyme that is part of N-glycosylation of proteins. N-linked glycans play a role in glycoprotein trafficking and cell signaling, and studies have shown that mutations in genes involved in the N-linked glycosylation pathway result in nervous system related disease [30]. *RFT1* was one of three genes that were also significantly differently expressed, in the same direction, in hypothalamic tissue from the same study population. *RBPMS* (RNA-binding protein with multiple splicing) contains a RNA recognition motif and can regulate transcriptional activity by binding to transcription factor c-Fos [31]. Studies in mammals have its expression localized to retinal ganglion cells [32]. *TOR3A* (torsin family 3 member A) belongs to a family of genes with a role in neurologic disease [33], as well as chaperone-like functions [34]. Lastly *SNAP23* (synaptosome associated protein 23kDa) plays an important role in functional regulation of postsynaptic glutamate receptors [35]. Glutamate is among the major excitatory neurotransmitters in the brain, and its relation to fear has been associated with variation in glutamate transporter translocation [36].

Gene ontology enrichment identified terms related to general cellular components as the cytoplasm and mitochondrion, and protein binding as a molecular function. The mitochondrion and cytoplasm association could possibly be related to previous results that have found changes in basal metabolic rate between the selection groups [17]. The KEGG pathway analysis did yield similar results, indicating an enrichment of genes in metabolic and signaling pathways. Of particular interest are the enrichments of genes in axon guidance and long-term potentiation (LTP), both related to behavioral processes. LTP is important in memory formation and the prefrontal cortex has a strong connection to the hippocampus where fear memory is processed [37]. As presented earlier, the gene *SNAP23* that was upregulated in low fear animals regulate postsynaptic glutamate receptors, the first step in LTP initiation. The disease association analysis results reflect the KEGG pathway results as several behavioral disorders and mitochondrial disease terms show enrichments. This indicate that many DE genes in S5 might be related to behavior.

The number of differentially expressed genes found is similar to that in hypothalamic expression differences in the same population of birds, but only 39 genes overlapped between the hypothalamic and cerebral hemisphere. When only genes remaining after FDR correction were taken into account, only three genes overlapped, *SPAG4*, *RFT1* and *ENSGALG00000016237*. The low amount of overlap might indicate tissue specific changes in association with the phenotypic changes. The function of *RTF1* has been discussed above, and with respect to *SPAG4*, this is a sperm-associated antigen-like protein localized in axoneme of sperm [38]. It is not clear what function this gene serves when expressed in the brain, but it is possible that it is somehow related to reproductive traits [18]. Another unconfirmed possibility is that the protein has a yet unknown function in the cytoskeleton of neural axons. The SPAG4 protein is also known as SUN domain-containing protein 5 (SUN5), and other SUN-domain proteins have been shown to be involved in neurogenesis and neuronal migration in mice [39, 40].

There were very few genes identified which overlapped with gene lists from other similar comparisons in other species and tissues (S1 Table). This does not support the theory of common genetic pathways in either hypothalamus or cerebral hemispheres underlying domesticated phenotypes in different species, but rather suggests that species-specific genetic mechanisms may be more plausible explanations. It remains unknown if there are any common genetic mechanisms explaining the cascade of phenotypic changes commonly observed.

Our studies of gene expression differences in this population of Red Junglefowl has so far concentrated on hypothalamus, as a center for control of fear and stress [18] and the cerebral hemispheres, an important region for, e.g., social behaviour. However, it is of course possible that other brain regions may be more important. For example, the telencephalon shows the greatest relative decrease in size in many mammals, including pig, sheep, rat and dog [5]. Likewise in birds, pigeon and turkey telencephalon size show a similar decrease in the domestic variant [41, 42]. Also in the present animal population, low fearful birds have a significantly smaller telencephalon than high fearful birds [Agnvall et al, unpublished data]. Hence, telencephalon should be worth exploring further for gene expression changes in this experimental chicken population.

In conclusions, we found a total of 417 significantly differentially expressed genes in the cerebral hemisphere comparing Red Junglefowl selected for five generations for high and low fearfulness towards humans. Twenty-two of these remained significant after FDR correction. Enrichment analysis showed that many of the affected genes and pathways are associated with behavioral processes. The results suggest that selection based on a single behavioral trait only (tameness) can change the transcriptome in only few generations.

## Supporting information

S1 Table Differentially expressed genes. Significantly differentially expressed genes before adjusting for multiple testing when comparing high fearful with low fearful S5 birds. The gene list cut off was set to *p* (unadjusted) < 0.01 which generated 417 significant genes. Table columns include microarray Probe ID (transcript ID), Ensembl Gene ID, annotated gene names, chromosomal probe position (chromosome and start position in base pairs), log fold change (FC) values, the unadjusted as well as adjusted *p*-values, overlap with previous studies, and biomaRt gene descriptions.
(XLSX)
